# Stimulation

**Published:** 1888-10

**Authors:** S. W. Dodds


### STIMULATION.

I believe the question of stimulation, as to what it is, and what its effect upon the human system, is one about which there is very little difference of opinion, in our ranks. It is well known that any substance taken into the system that cannot be utilized, has to be thrown out; and that those substances which are largely expelled through that great depurating surface, the skin, are of the class known as stimulants. Whiskey is a good example of this class. So are the other alcoholic beverages; they are all more or less stimulating. That feeling of exhilaration which is experienced soon after taking them, is nothing more or less than the rallying of the vital forces to get rid of an intruder—a foe in the realm of life.

After this expulsive effort is over—it may take a few hours, more or less—there is a feeling of exhaustion or prostration; nature is tired out from over-exhaustion, and seeks repose. Then it is that the victim of this "strengthening" delusion again seeks his bottle, or his drug; repeats the stimulating process, and this is again followed by a period of collapse.

And thus it is that thousands of the human race are cheated, and are gradually using up the vitality that nature has given them, and which is needed to carry on the life work properly. By and by the kidneys or other depurating organs break down (are worn out) and an incurable affection puts an end to life. Or, if these organs still hold out, the nerves—exhausted by their continued tax upon them—begin to give out, and the general health suffers. Or, the very blood itself may begin to disorganize; clots may form in the brain or elsewhere, and paralysis sets in; it may be local or general. Or, escaping this, the brain may congest and a fit of apoplexy come suddenly, and death be the result. How many of our people are suffering in this very way—many from the habitual use of stimulants, or from other causes combined.

Physiologically speaking, whatever enters the circulation either builds up or destroys; so that all things may, in this sense, be resolved into foods and poisons. It is a lamentable fact, that much that is taken as food or drink, is not food, in any sense; instead of nourishing it wastes. Alcohol or tobacco, bad as they are known to be in every way, cannot be held responsible for all the mischief done. Even tea and coffee, with all their injurious effects, are not the only other thieves that steal away our life capital, leaving us poor and wretched. Neither does the great army of spices and condiments—every one of them mischievous—complete the rank and file of the "enemy." These do their share, it is true, in perverting natural taste, exhausting the supply of digestive fluids, congesting the mucous surfaces, wearing out the liver and kidneys paralyzing the nerve centers, and finally ending up with confirmed dyspepsia, and chronic invalidism. Thousands to-day are suffering, and are searching in vain for some "remedy" which shall make everything lovely ; quite forgetting that an infringement of Nature's laws—which are God's laws—is not to be met in this way.

But I must leave the rest of this subject for after consideration.—S. W. Dodds, M. D , in *The Journal of Hygeio-Therapy.*

